# Visualizing extracellular vesicle biogenesis in gram-positive bacteria using super-resolution microscopy

**DOI:** 10.1186/s12915-022-01472-3

**Published:** 2022-12-05

**Authors:** Dokyung Jeong, Min Jeong Kim, Yejin Park, Jinkyoung Chung, Hee-Seok Kweon, Nae-Gyu Kang, Seung Jin Hwang, Sung Hun Youn, Bo Kyoung Hwang, Doory Kim

**Affiliations:** 1 Department of Chemistry, Hanyang University, Seoul, 04763 Republic of Korea; 2 Electron Microscopy Research Center, Korea Basic Science Institute, Cheongju, 28119 Republic of Korea; 3 R&D Center, LG H&H Co., Ltd, Seoul, 07795 Republic of Korea; 4 Research Institute for Convergence of Basic Science, Hanyang University, Seoul, 04763 Republic of Korea; 5 Institute of Nano Science and Technology, Hanyang University, Seoul, 04763 Republic of Korea; 6 Research Institute for Natural Sciences, Hanyang University, Seoul, 04763 Republic of Korea

**Keywords:** Extracellular vesicles, Super-resolution microscopy, STORM, Gram-positive bacteria

## Abstract

**Background:**

Recently, bacterial extracellular vesicles (EVs) have been considered to play crucial roles in various biological processes and have great potential for developing cancer therapeutics and biomedicine. However, studies on bacterial EVs have mainly focused on outer membrane vesicles released from gram-negative bacteria since the outermost peptidoglycan layer in gram-positive bacteria is thought to preclude the release of EVs as a physical barrier.

**Results:**

Here, we examined the ultrastructural organization of the EV produced by gram-positive bacteria using super-resolution stochastic optical reconstruction microscopy (STORM) at the nanoscale, which has not been resolved using conventional microscopy. Based on the super-resolution images of EVs, we propose three major mechanisms of EV biogenesis, i.e., membrane blebbing (mechanisms 1 and 2) or explosive cell lysis (mechanism 3), which are different from the mechanisms in gram-negative bacteria, despite some similarities.

**Conclusions:**

These findings highlight the significant role of cell wall degradation in regulating various mechanisms of EV biogenesis and call for a reassessment of previously unresolved EV biogenesis in gram-positive bacteria.

**Supplementary Information:**

The online version contains supplementary material available at 10.1186/s12915-022-01472-3.

## Background

The extracellular vesicle (EV) is produced by many organisms, including eukaryotes, archaea, and bacteria, and acts as a universal vesicular transport system by secreting proteins, molecules, polysaccharides, and other factors [1, 2]. Since EVs were first reported in *Escherichia coli* in the 1960s as the first bacterial EVs, bacterial EVs have been suggested to play crucial roles in various biological processes, including phage infection, bacterial communication, and the transport of genes, virulence factors, and cellular metabolites [3–5]. Moreover, they have been reported to play an important role in carbon cycling in the marine ecosystem and protecting biofilm cells from antibiotics by being an integral constituent of the biofilm matrix [6, 7]. Due to the valuable roles of bacterial EVs in various biological processes, they are expected to have great potential not only for developing cancer therapeutics but also for applications in biomedicine, including vaccines [8–11]. For example, bioengineered bacterial membrane vesicles have been demonstrated to target and kill cancer cells by delivering small interfering RNA against tumor markers [8]. Also, several intrinsic properties of bacterial membrane vesicles make them promising vaccine candidates, such as various surface antigens in a native conformation, immunogenicity, self-adjuvation, and uptake by immune cells [10]. Moreover, they are known to be versatile as they can be bioengineered to express any chosen antigen and manipulated to reduce their endotoxicity [9]. Therefore, research attention on the bacterial EV has increased substantially over the past decade.

Until now, studies on bacterial EVs have mainly focused on the outer membrane vesicles (OMVs) released from gram-negative bacteria, which originate from the outer membrane of gram-negative bacteria under the control of membrane blebbing [2, 5]. In contrast, EV production by gram-positive bacteria has been overlooked due to the absence of an outer membrane and the inference that the thick peptidoglycan cell wall in gram-positive bacteria precludes the release of EV as a physical barrier [2, 5]. However, recent studies have reported biologically active EVs from gram-positive bacteria, stimulating research on the biogenesis of EVs in gram-positive bacteria [12]. Although the mechanisms underlying EV formation in gram-positive bacteria remain unclear, several models for EV biogenesis in gram-negative bacteria have been proposed, including increased turgor pressure from the periplasmic space, repulsion between charged lipopolysaccharide molecules, and depletion of the peptidoglycan and outer membrane linkage [2, 5, 11]. Given the different cell wall compositions in gram-positive and gram-negative bacteria, they could differ in the routes for EV formation.

Currently, the observation and quantification of EV formation in gram-positive bacteria remains challenging due to the technical difficulties associated with imaging the ultrastructure of small-sized EVs (20–400 nm in diameter) within the diffraction-limited area using conventional light microscopy (LM) techniques [5]. Although electron microscopy (EM) can provide higher resolution than LM, the molecular-specific labeling efficiency is limited in EM images [13–15]. These difficulties can be overcome using recently developed super-resolution fluorescence microscopy, which enables nanoscale imaging with high molecular specificity [16–19].

In this study, the mechanisms underlying EV biogenesis in gram-positive bacteria were investigated using super-resolution stochastic optical reconstruction microscopy (STORM). For comparison, various EM techniques were also performed, including scanning electron microscopy (SEM), transmission electron microscopy (TEM), and correlative STORM and EM. These approaches were used to reveal ultrastructural changes in the EV at the cell envelope during the maturation process via observations of N-acetylglucosamine, protein A, enterotoxin B, and the membrane at the nanoscale. These findings indicate the existence of diverse mechanisms for EV biogenesis in gram-positive bacteria, which have not yet been resolved using conventional microscopy.

## Results

### Nanoimaging of EVs from gram-positive bacteria

**Fig. 1:**
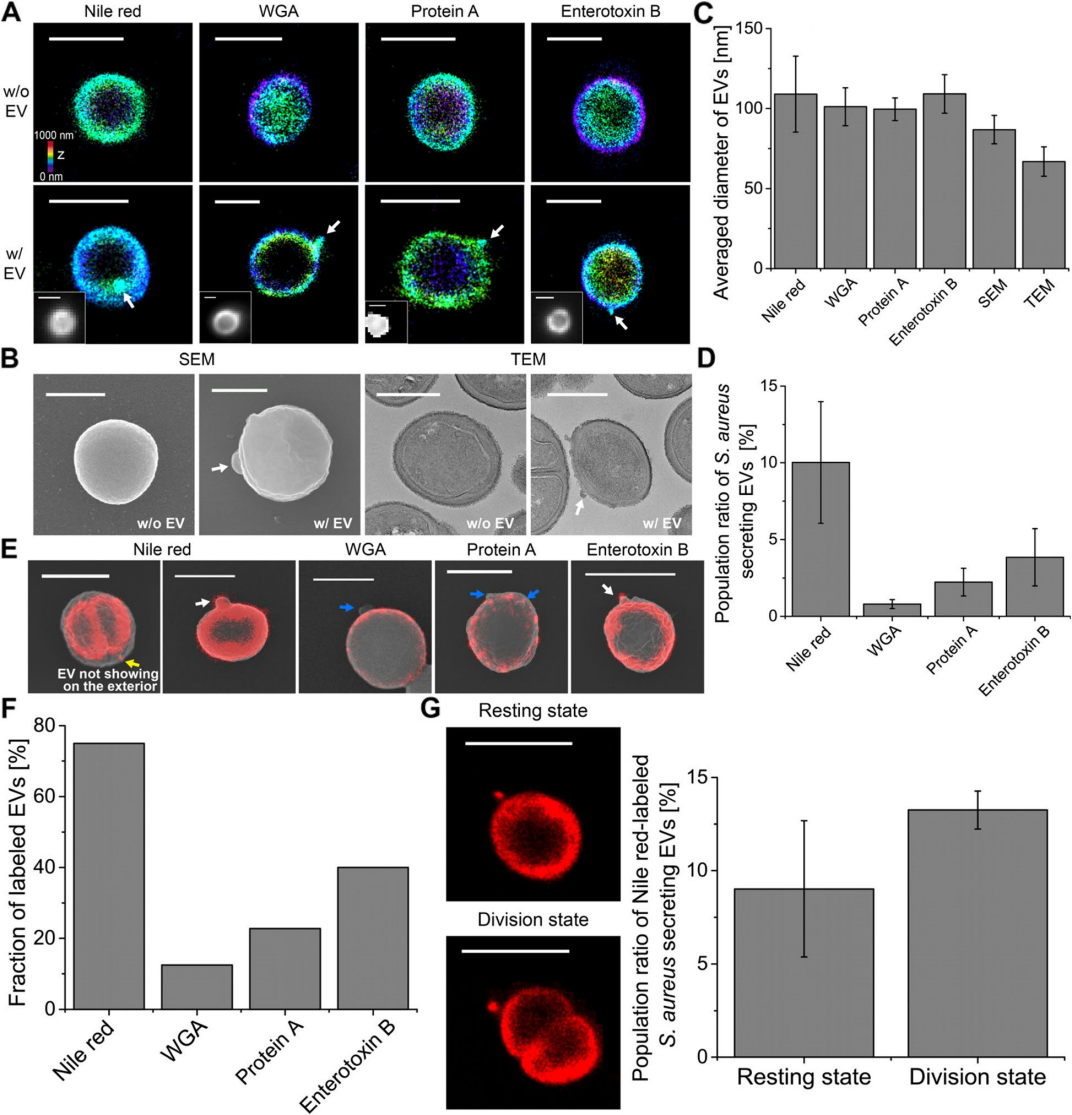
Super-resolution images of extracellular vesicles (EVs) from gram-positive bacteria. **A** 3D STORM images of *S. aureus* without EVs (top) and with EVs (bottom). *S. aureus* was labeled with Nile red, WGA, anti-protein A, or anti-enterotoxin B. Inset: Diffraction-limited fluorescence images of the same area. **B** SEM (left) and TEM (right) images of *S. aureus* without EVs and with EVs. **C** The average diameter of EVs measured from STORM (Nile red, WGA, protein A, and enterotoxin B), SEM, and TEM images (mean±SD; *n*=11–75). **D** Comparison of the population ratio of *S. aureus*-secreting EVs observed from STORM images of the sample labeled with Nile red/WGA/anti-protein A/anti-enterotoxin B (mean±SD; *n*=700–1400). **E** Correlative STORM and SEM images of EVs labeled with Nile red, WGA, anti-protein A, or anti-enterotoxin B. White arrow: EV observed both from STORM and SEM images. Yellow arrow: EV observed from the Nile red image, which was not shown in the SEM image, implying a membrane vesicle located inside the peptidoglycan layer before the budding process through the cell wall. Blue arrow: EV observed from the SEM image, which was not shown in the STORM image of the cell wall, implying EVs without the peptidoglycan layer. **F** Fraction of labeled EVs with Nile red, WGA, anti-protein A, or anti-enterotoxin B among the EVs shown in SEM images from correlative STORM and SEM images (*n*=8–22). **G** Representative STORM images and the population ratio of Nile red-labeled *S. aureus*-secreting EVs in the resting state or in the division state. (mean±SD; *n*=170–540) Scale bars: 1 μm in **A**, **E,** and **G** and 500 nm in **B**

We first investigated whether EVs could be observed in gram-positive bacteria using super-resolution STORM. We chose *Staphylococcus aureus* and *Staphylococcus epidermidis* because they are common members of the normal human microbiota. *S. aureus* and *S. epidermidis* were first labeled with Nile red dye to observe their membrane structures and then imaged using 3D STORM imaging. From the single-molecule localization distribution analysis and the xy-cross-section images of Nile red-stained bacteria, we confirmed the nanoscale resolution of this imaging method (Additional file 1: Fig. S1). From the STORM images of Nile red-stained *S. aureus*, we also observed the septum structure in the dividing bacteria, implying successful membrane labeling of the bacteria by Nile red dye molecules (Additional file 1: Fig. S2). As shown in Fig. 1A, Additional file 2: movie 1, and Additional file 3: movie 2, most *S. aureus* isolates (~90%) did not have EVs with spherical shapes. In contrast, ~10% of *S. aureus* isolates were shown to have EVs on their surfaces, which has not been previously observed by diffraction-limited LM owing to their small size (~60–150 nm in diameter).

To investigate whether they are Nile red precipitates or real EVs, we performed a single-molecule distribution analysis of the Nile red-only sample without EVs under our experimental conditions (Additional file 1: Fig. S1B). Any precipitate would result in noticeable changes in the single-molecule distribution, such as large positional variations with large FWHM values or an asymmetric distribution. However, the positional variation statistics within each nanocluster resulted in an FWHM (localization uncertainties) of 26–28 nm, in agreement with what is typically achieved for a single Nile red molecule in STORM, implying that aggregation was prevented under our optimized conditions and the observed nanoclusters were real EVs, not Nile red precipitates. We also tested other membrane dyes, including CellMask and CellBrite. As shown in Additional file 1: Fig. S3, we observed EVs from the staining of CellMask and CellBrite similar to Nile red-stained EVs. From the quantitative analysis of EV diameter measurement, they showed spherical shapes with a similar diameter (99–108 nm), regardless of the type of membrane dye.

To further confirm that the observed small-sized spherical particles were indeed EVs, we purified the EVs from cultured *S. aureus* and then performed STORM imaging after Nile red labeling. As shown in Additional file 1: Fig. S4, the Nile red-labeled EVs from purified samples also exhibited a spherical shape with a size similar to that of the observed nanoparticles in Fig. 1A, confirming that the observed small nanoparticles on the bacteria are real EVs. Thus, it suggests that our super-resolution imaging method can be useful in identifying EVs by confirming both the particle morphology and membrane composition, which have not been simultaneously analyzed at the single EV level from any conventional EV detection methods, such as nanoparticle tracking analysis, SEM/TEM imaging, or proteomic analysis. We also found that EVs were also observed in the STORM images of the Nile red-stained *S. epidermidis* (Additional file 1: Fig. S5). However, only a few *S. epidermidis* (~2.5%) were observed to have EVs on their surfaces compared to *S. aureus* (~10%), which may imply the distinct roles of EVs in different strains.

Next, we investigated whether EVs could also be observed in the cell wall of *S. aureus* after staining. We used wheat germ agglutinin (WGA) and anti-protein A to label the bacterial cell wall. WGA binds to N-acetylglucosamine in the outer peptidoglycan layer of gram-positive bacteria [20], and protein A is known to be a cell wall-associated protein [21, 22]. Since WGA can bind only to the peptidoglycan layer, hollow and spherical shapes of bacteria were observed from 3D STORM images, whereas the septum in the dividing bacteria was not observable in the cell wall images (Fig. 1A, Additional file 1: Fig. S2). Interestingly, we also observed EVs in the STORM images of the surface of *S. aureus* labeled with WGA and anti-protein A; these EVs were not resolved in the diffraction-limited fluorescence images. Next, we immunolabelled enterotoxin B in *S. aureus*. Enterotoxins are known to be one of the most common causes of food poisoning due to uncontrolled T cell activation, followed by toxic shock and death [23]. Since it has been reported that enterotoxin A and B are bound to microvillus membrane vesicles in vitro, we expected to observe EVs from bacterial enterotoxin images [23]. As shown in Fig. 1A, EVs were observed on the surface of *S. aureus* in the corresponding enterotoxin B-stained STORM images. The observed staining patterns in bacteria were similar to those of the peptidoglycan layer rather than the cytoplasmic membrane because the septum in the dividing bacteria was not stained with the enterotoxin B immunolabel, implying that the immunolabeled-enterotoxin B was located in the cell wall of gram-positive bacteria. This observation is also supported by a previous study demonstrating that enterotoxin B is associated with the cell wall after its precursor form is processed and released from the membrane [24]. This previous study also reported that the cell wall-associated enterotoxin B level decreased proportionately as the extracellular fraction of enterotoxin B increased during a pulse-chase experiment [24]. The observations of our STORM images and the findings of this previous study suggest that enterotoxin B appears to be released into the extracellular environment in the form of EV. Thus, this secreted virulence factor may be transferred from the cell wall of gram-positive bacteria to EVs. We also performed SEM and TEM imaging to observe EVs released from *S. aureus*. As shown in Fig. 1B, some *S. aureus* cells were observed to have EVs on their surfaces in the SEM and TEM images, similar to the STORM images.

Using the various EV images, we analyzed the size of the observed EVs. First, we compared the membrane diameters measured from the STORM images of EVs secreted from bacteria and the purified EVs. The average membrane diameters measured from EVs on bacteria and the purified EVs were ~113 and ~107 nm, respectively, confirming that the observed small nanoparticles on bacteria are real EVs (Additional file 1: Fig. S4B). From the diameter measurement of EVs, we found that the averaged diameters of EVs observed from STORM images of a membrane and a peptidoglycan layer range between 100 and 110 nm, which is within the reported EV size range [5] (Fig. 1C). As expected, the EVs observed in the SEM and TEM images appeared smaller than those observed in the STORM images owing to the absence of a tag and the shrinkage effect during the dehydration step in EM. Interestingly, we observed at least two populations based on the size in the STORM images, SEM images, and TEM images, implying the heterogeneity of EVs generated from different biogenesis mechanisms (Additional file 1: Fig. S6). Next, we analyzed the EV production rates of *S. aureus* from each type of image. Interestingly, the STORM images of the bacterial membrane exhibited a higher rate of EV production (~10%) than the STORM images of the cell wall (~1 and ~2% from WGA and protein A images, respectively) (Fig. 1D). Furthermore, the STORM images of enterotoxin B-labeling exhibited a higher rate of EV production (~4%) than the STORM images of the cell wall. These results suggest various compositions of EV for the membrane and peptidoglycan layers. Such variations in the composition of EV have not been resolved at a single EV level with conventional EV analysis techniques, emphasizing the importance of our nanoscale imaging-based analysis technique. Meanwhile, it was noted that fewer EVs were observed from the SEM images compared with the STORM images of the bacterial membrane. This could be because some EVs observed from membrane images had not yet budded out to the outside of the bacterial outer surface and were still located inside the peptidoglycan layer. Thus, they were not observable in the topography of the SEM images. The different production rates observed from various images prompted us to investigate the composition and location of EVs.

To confirm the composition of EVs, we performed recently developed correlative STORM and SEM imaging (Fig. 1E, Additional file 1: Fig. S7) [25, 26]. We performed SEM imaging after STORM imaging and EM sample preparation, similarly to a previously reported method [25]. Interestingly, most EVs (~82%) observed in the SEM images do not show the peptidoglycan layers, whereas only a small number of EVs (~18%) showed peptidoglycan layers (Fig. 1F). We also observed Nile red-stained membrane vesicles located inside the peptidoglycan layer in bacteria, which are not shown in the SEM images (Fig. 1E). As they appear to wait for the subsequent budding process through the cell wall, we refer to them as EV precursors. We found that its population changed in the same way as the EV production rate under various stresses such as temperature, osmotic stress, and the bacterial growth phase. For example, more membrane vesicles located inside the peptidoglycan layer were observed in the cultures incubated at 30 °C, under low salt conditions, or in a division state, in a similar fashion to the released EVs (Additional file 1: Fig. S8). Since it is difficult to imagine other bacterial organelles in a spherical shape located between the peptidoglycan layer and the cytoplasmic membrane (i.e., IWZ: inner wall zone), the observed membranous particles inside the peptidoglycan layer appear to be EV precursors. Furthermore, some bacteria with EVs on their surface showed a lower labeling density of WGA or protein A, implying a degraded cell wall structure during the EV release process. A weakened cell wall structure has been reported to be often observed in gram-positive bacteria during the division phase [27, 28]. Thus, we next quantified the EV production rate depending on the bacterial division phase. Interestingly, we found that bacteria in the division phase with septum formation showed a higher rate of EV production (Fig. 1G). Collectively, EV biogenesis appears to involve the cell wall lysis process for bacteria to release budding EVs.

### Composition of EVs generated from gram-positive bacteria

We successfully used specific labeling methods for cell membranes and cell walls of gram-positive bacteria to perform multi-color STORM imaging for observing the cell membrane and cell wall simultaneously during the EV biogenesis process. Although several proteomic analyses of membrane vesicles from purified EV samples have been previously used to identify the composition of EVs, there is a chance of contamination by cell fragments or debris, such as protein aggregates, using these methods [29]. However, this limitation can be overcome by multi-color STORM imaging of EV-producing gram-positive bacteria because this method can be used to observe EVs in situ. This imaging method also allows us to observe the composition of individual EVs without contamination problems as opposed to previous ensemble measurements.

**Fig. 2:**
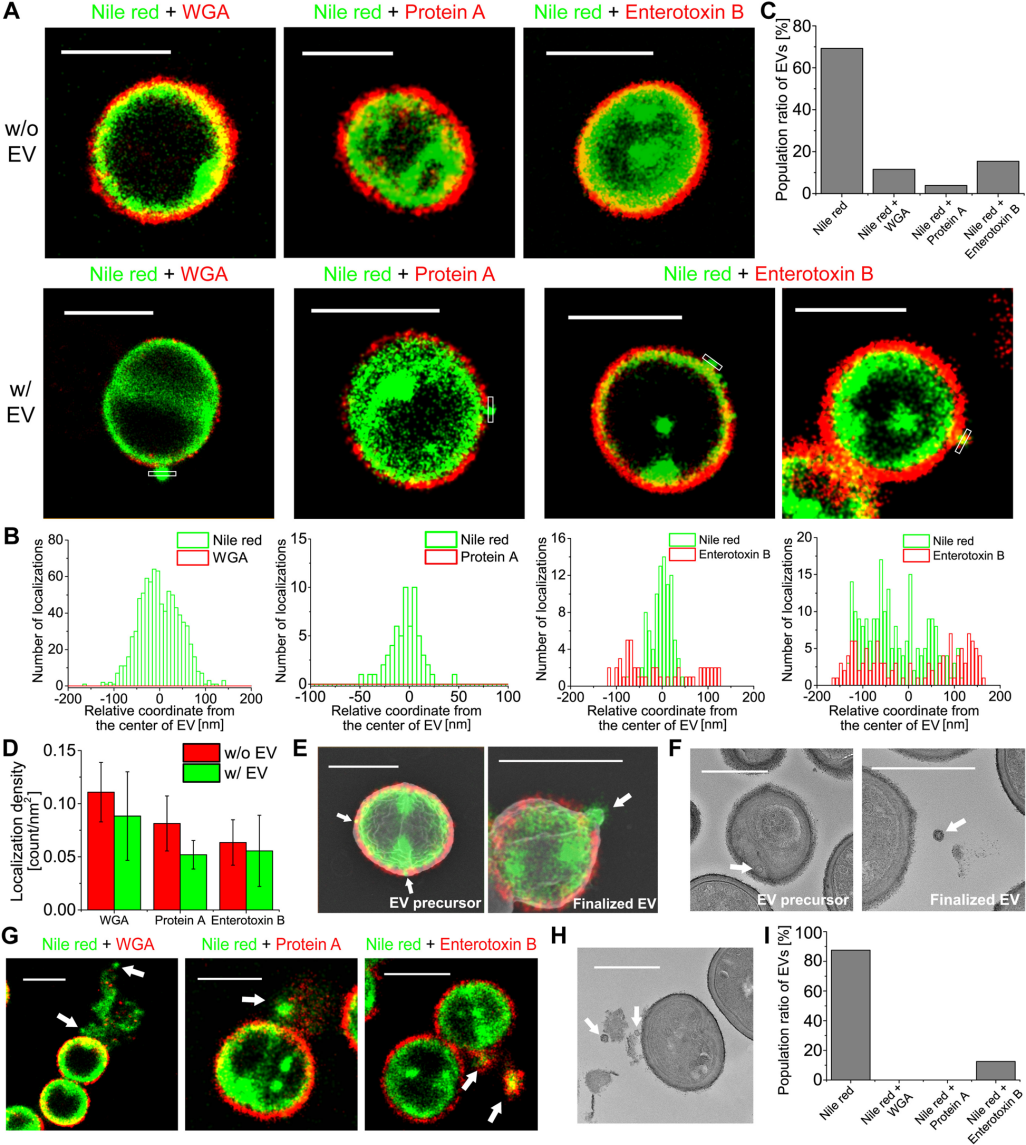
Multi-color STORM images for the various compositions of EVs in gram-positive bacteria. **A** Representative multi-color STORM images of *S. aureus* without EVs (top) and with EVs (bottom). Red: WGA, protein A, or enterotoxin B. Green: Nile red. **B** Transverse profiles of localizations corresponding to regions boxed in white in (**A**). Green bars: Localization frequency measured from the STORM image of Nile red. Red bars: Localization frequency measured from the STORM image of WGA, protein A, or enterotoxin B. **C** Population ratio of EVs displaying one color (Nile red only) or two colors (Nile red and WGA/ protein A/enterotoxin B) measured from the EVs on the bacterial surface (mean±SD; *n*=~30). **D** Comparison of averaged localization density of WGA, protein A, and enterotoxin B of EV-producing *S. aureus*. **E** Representative correlative multi-color STORM and SEM images of *S. aureus* with EV precursors or finalized EVs. **F** Representative TEM image of *S. aureus* with EV precursors or finalized EVs. **G** Representative multi-color STORM images of *S. aureus* with EVs produced after the explosive cell lysis event. Red: WGA, protein A, or enterotoxin B. Green: Nile red. **H** Representative TEM image of *S. aureus* with produced EVs after the explosive cell lysis event. **I** Population ratio of EVs displaying one color (Nile red only) or two colors (Nile red and WGA/ protein A/enterotoxin B) measured from the STORM images of the released EVs after the explosive cell lysis event (*n*=~20). White arrows: observed EVs. Scale bars: 1 μm in **A**, **E**, and **G** and 500 nm in **F** and **H**

Using multi-color STORM imaging for various combinations of proteins, we observed the nanostructures of the inner plasma membrane and outer peptidoglycan layers in *S. aureus*, as shown in Fig. 2A and Additional file 1: Fig. S9. Interestingly, most EVs only showed a membrane layer, whereas a few EVs showed a double layer of WGA (or protein A) and membrane, as shown in Fig. 2B,C. This is consistent with the population ratios observed from the correlative single-color STORM and SEM images shown in Fig. 1F. This is also consistent with the size heterogeneity shown in Additional file 1: Fig. S6, implying the existence of various mechanisms for EV biogenesis. Interestingly, enterotoxin B exhibits different localization from WGA and protein A. Although we demonstrated that enterotoxin B is the cell wall-associated protein in *S. aureus*, its density did not decrease in the EV-producing bacteria, which is different from WGA and protein A (Fig. 2D). This implies that enterotoxin B remains the outermost layer of bacteria during cell wall lysis and is then transferred to the released EVs as a virulence factor.

Next, the correlative multi-color STORM and SEM imaging was performed to differentiate the EV precursors located inside the peptidoglycan layer in bacteria from the released EVs. The results showed that the EV precursors were located inside the peptidoglycan layer in bacteria, different from the location of finalized EVs (Fig. 2E, Additional file 1: Fig. S10). These EV precursors are likely resulted from the membrane blebbing, as known for the OMV released from gram-negative bacteria. As expected, the EV precursors observed in the multi-color STORM images were not observed in the SEM images, most likely due to still being encapsulated by the outermost cell wall. These EV precursors located in the IWZ were also observed from the TEM images (Fig. 2F). These EV precursors resulted from the membrane blebbing appear to wait for the subsequent budding process through the cell wall. We also observed an explosive cell lysis event in bacteria, which is known to act as a mechanism for EV production in gram-negative bacteria [30]. Interestingly, a relatively large amount of membrane fragments were spread over a wide area, whereas the cell wall fragments were observed to scatter after the explosive cell lysis (Fig. 2G). As EVs consisting of the membrane were often observed within these cell debris, the secreted membrane fragments appeared to form EVs rapidly, similar to the formation in gram-negative bacteria. EV formation after cell lysis was also observed in the TEM images (Fig. 2H). Interestingly, enterotoxin B exhibited higher labeling rate for EVs produced by explosive cell lysis, in contrast to the protein A and WGA patterns (Fig. 2I). This suggests that enterotoxin B can be transferred to EVs with membrane fragments as a major virulence factor after the explosive cell lysis event. Collectively, these findings highlight the variety of EV compositions produced by different mechanisms, as observed in the multi-color STORM images.

### EV biogenesis mechanisms for gram-positive bacteria

**Fig. 3:**
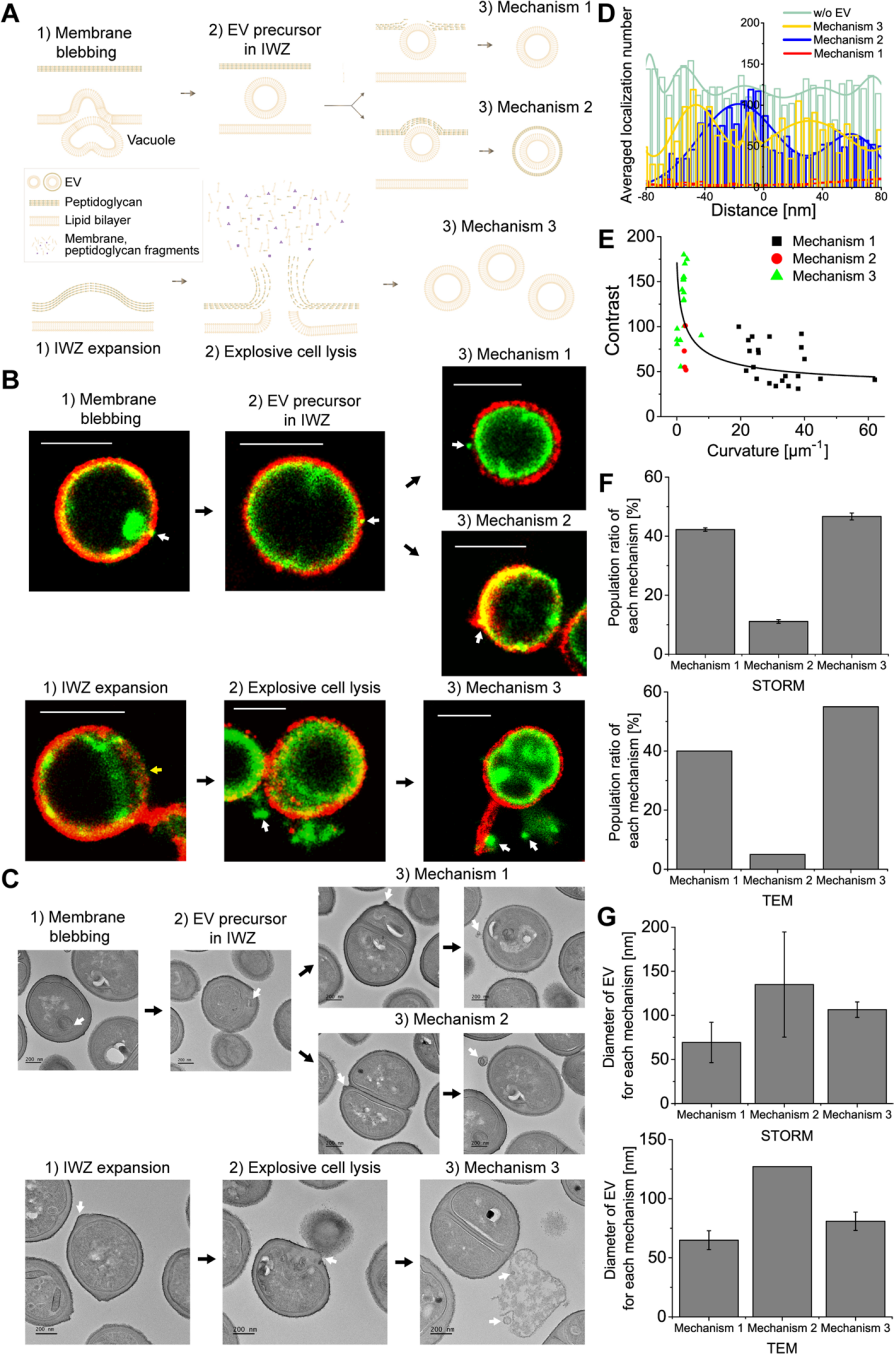
Proposed EV biogenesis mechanisms in gram-positive bacteria. **A** Proposed models for EV biogenesis in gram-positive bacteria. **B, C** Representative (**B**) multi-color STORM and (**C**) TEM images of *S. aureus* with EVs for each mechanism. Red: WGA. Green: Nile red. Yellow arrow: expanded IWZ. **D** Comparison of the cell wall density for each mechanism observed from STORM images. Averaged localization number in the cell wall was measured as the cell wall density from STORM images. **E** Relationship between the contrast and the curvature of the cell wall measured from TEM images. The fitting line obtained by the exponential function shows an inverse relation. **F** Population ratio of *S. aureus* producing EVs by each mechanism observed from multi-color STORM and TEM images (mean±SD; *n*=~1000 from STORM images, *n*=~80 from TEM images). **G** Comparison of the diameter of EVs produced by each mechanism observed from STORM and TEM images (mean±SD; *n*=~30). White arrows: observed EVs. Scale bars: 1 μm in **B** and 200 nm in **C**

Based on the observation and measurement of EVs in multi-color STORM and TEM images, EV biogenesis in gram-positive bacteria appeared to occur either through membrane blebbing or explosive cell lysis, similar to the occurrence in gram-negative bacteria (Fig. 3A).

Membrane blebbing was observed in the STORM images of Nile red-stained EVs (Fig. 3B, Additional file 1: Fig. S11). Interestingly, a vacuole-like structure composed of a high-density lipid was often observed in Nile red images immediately below the budding site inside the cytoplasmic membrane, most likely acting as a readily available membrane source (Fig. 2A,B). Such a vacuole generation has been reported to frequently occur during the bacterial cell enlargement inside the cytoplasm of gram-positive bacteria [31]. Then, the blebs seemed to first remain between the cytoplasmic membrane and peptidoglycan layer as EV precursors after being pinched off from the plasma membrane, in contrast to previously suggested EV biogenesis models for gram-positive and gram-negative bacteria. In previous studies, the cytoplasmic membrane has been described to extrude through the hole in the peptidoglycan layer for EV generation in gram-positive bacteria without the precursor state, based on the observation of membrane blebbing after lysin treatment [32]. In contrast, these extruding membrane bubbles were not observed through the thick peptidoglycan layer in our STORM and TEM images. Instead, many membrane vesicles remained in the IWZ, which is the region between the cytoplasmic membrane and the peptidoglycan layer, after being pinched off from the cytoplasmic membrane. Such membrane vesicles were not observed in the SEM images of correlative STORM and SEM imaging as shown in Fig. 1E because they were still encapsulated inside the peptidoglycan layer. This discrepancy between previously reported observations and the observations in this study is most likely due to the different cell wall conditions arising from the lysin treatment used in the previous study. Removal of the outer cell wall zone upon lysin treatment in the previous study allowed the membrane bleb to extrude easily through the peptidoglycan layer. In contrast, the native bacteria without lysin treatment in the present study seemed to have a relatively thicker peptidoglycan layer as a barrier, preventing membrane bleb from extruding before being pinched off from the membrane. The EV precursors located in the IWZ appeared to wait for the degradation of the peptidoglycan layer. The IWZ is composed mostly of soluble low-density constituents, allowing vesicles to expand within it [33]. We found that most of the EV precursors were released after local cell wall lysis (mechanism 1), whereas a few of them were released by being encapsulated by the remaining peptidoglycan layer (mechanism 2) (Fig. 3B,C). Since the latter is occasionally observed (~11% from STORM images), this mechanism may have been difficult to observe in previous studies. Because these two mechanisms appear to involve the cell wall lysis step, the cell wall contrast near the EV-releasing area was analyzed in the STORM and TEM images. As shown in Fig. 3D, local cell wall degradation was observed near the EV-locating area for mechanisms 1 and 2 in both the STORM and TEM images. Although cell wall density was slightly higher at the budding site in mechanism 2, the surrounding cell wall appeared to degrade to aid in the release of EV from the cell wall via this mechanism.

EV biogenesis through explosive cell lysis has also been frequently observed in multi-color STORM images (mechanism 3). In relation to this mechanism, expanded IWZ was observed in the corresponding TEM images, most likely due to the increased osmotic pressure in this zone (Fig. 3B, Additional file 1: Fig. S12). The turgor pressure exerted from this zone pushes the cell wall, resulting in low-curvature blebbing of the peptidoglycan layer. At this point, the turgor pressure exerted from inside the membrane appeared to be larger than the osmotic pressure in the IWZ, resulting in the bending of the peptidoglycan layer instead of the cytoplasmic membrane at the budding site. When we analyzed the density (contrast) and curvature of the cell wall at this budding site, relatively higher contrast and a lower curvature of the cell wall were observed in mechanism 3 compared to mechanisms 1 and 2, implying that less cell wall lysis occurred in mechanism 3 by allowing the expansion of the IWZ (Fig. 3E). This expanded IWZ ultimately ruptured due to cell wall damage and increased turgor pressure, as observed in the STORM and TEM images. During this explosive cell lysis, the formation of multiple EVs was observed in both the STORM and TEM images. EVs in this mechanism seemed to be composed of a membrane without the peptidoglycan layer as shown in Fig. 2I, although cell wall fragments were also secreted during the cell lysis process. This suggests that the amount of cell wall fragments released by explosive cell lysis is insufficient for forming the peptidoglycan layer for EVs since the cell wall was already degraded prior to the explosive cell lysis event. Interestingly, we also found that the EVs produced after cell lysis process were hardly observed from SEM images because they were covered by the released cell debris during explosive cell lysis (Additional file 1: Fig. S13). Although the EVs produced by mechanism 3 were not directly observed from the SEM images, the spherical membrane vesicles observed after explosive cell lysis in STORM images are highly likely to be real EVs as EVs produced after explosive cell lysis were frequently observed not only from gram-negative bacteria in previous studies, but also from our TEM images. This suggests that our STORM imaging method can be a better tool for observing EVs produced by explosive cell lysis than SEM imaging.

Among these, mechanisms 1 and 3 were found to be the major mechanisms for EV biogenesis in gram-positive bacteria (Fig. 3F). Although mechanism 2 occurred rarely, the EVs produced by this mechanism were observed in both the STORM and TEM images. As expected, the EVs produced by mechanism 2 were larger than those produced by mechanism 1 (Fig. 3G). Interestingly, the EVs produced by mechanism 3 also exhibited a relatively larger size than those produced by mechanism 1. This could be due to the growth of EV precursors within the IWZ in mechanism 1 being limited by the width of the expanded IWZ. In contrast, the growth volume for EV production is not limited during the explosive cell lysis in mechanism 3, as the EVs are formed after secretion from the IWZ region. Therefore, we could confirm that the size heterogeneity of EVs is resulted from various EV biogenesis mechanisms in gram-positive bacteria, as expected. Collectively, various EV biogenesis mechanisms in gram-positive bacteria could be investigated by using the multi-color STORM images.

## Discussion

We used multi-color STORM and correlative super-resolution microscopy to elucidate EV biogenesis in gram-positive bacteria by resolving the ultrastructure of EVs produced during their formation processes. This has not been previously investigated in-depth compared to the investigation of gram-negative bacteria. Although gram-negative bacteria have different cell wall structures, we observed similar EV generation mechanisms in gram-positive bacteria, including membrane blebbing and explosive cell lysis.

Membrane blebbing was frequently observed in *S. aureus* and *S. epidermidis* in the corresponding STORM images, which was not previously resolved in diffraction-limited optical images. This mechanism resembled outer membrane blebbing during the EV biogenesis observed in gram-negative bacteria. However, the different structures of their outer layers affected the detailed process of EV production via membrane blebbing. For example, cell wall degradation is not a prerequisite in gram-negative bacteria because EVs are produced through outer membrane blebbing. Instead of cell wall degradation, EV production requires structural changes in the cell envelope, such as a decrease in cross-linking proteins between the outer membrane and peptidoglycan layer or the intercalation of molecules in the membrane at specific regions of the cell envelope [29]. In contrast, in this study, gram-positive bacteria were found to require a cell wall degradation process to release EV via the membrane blebbing mechanism due to the thickness of the peptidoglycan layer as the outermost layer. Degradation of the cell wall was observed before EV release by imaging WGA and protein A at the nanoscale level. As a result of the cell wall degradation experienced by the bacteria in the division phase, a higher rate of EV production was observed. Another distinct phenomenon during EV biogenesis in the membrane blebbing mechanism of gram-positive bacteria was the EV precursor located in the IWZ, which has not been previously observed in gram-negative bacteria. The bacteria were expected to grow further in the IWZ because it is composed of soft materials, which allow EV precursors to expand within it. Once the cell wall was ready post-cell wall degradation, EV precursors appeared to be released either through the pores (mechanism 1) or via cell wall blebbing (mechanism 2), depending on the condition of the cell wall. During this process, we suspect that cell wall-modifying enzymes may degrade the cell wall, increasing the cell wall pore size, as demonstrated in previous studies [2]. Although the increased pore size in the cell wall would facilitate the EV release (mechanism 1), the EV precursors were occasionally found to be released via cell wall blebbing (mechanism 2). After the degradation of the cell wall, the loosened peptidoglycan layer may be easily blebbed by the forces exerted by the EV precursors, resulting in the encapsulation of the EV precursors by the peptidoglycan layer during the process of EV release. Thus, double layers of EVs consisting of the cytoplasmic membrane and peptidoglycan layer were observed, which, to the best of our knowledge, has not been reported to date (mechanism 2). Although this mechanism (mechanism 2) rarely occurs, the EVs produced by this mechanism are expected to play a distinct role compared with those produced by other mechanisms because they have an additional peptidoglycan layer. For example, the peptidoglycan layer in EVs produced by mechanism 2 may prevent osmotic lysis of EVs, similar to its role as a physical barrier to bacteria. Because EVs consisting only of a membrane layer would experience the net flow of free water into the EVs due to the concentrated proteins, molecules, polysaccharides, and other factors inside the EVs, they would easily explode by this osmotic pressure without a strong peptidoglycan layer. Another possibility is that EVs transport essential cell wall-associated components through the peptidoglycan layer. Mechanisms 1 and 2 may be regulated by the cell wall condition, most likely resulting from the cell wall-modifying enzyme.

EV formation through explosive cell lysis has also been frequently observed in gram-positive bacteria, similar to that in gram-negative bacteria. Before the explosion, an expanded IWZ was first observed, most likely due to the increased osmotic pressure through the degraded porous cell wall. The blowing region in the IWZ finally exploded, perhaps in response to external stress, secreting various proteins, such as membrane fragments, cell wall fragments, and virulence factors. During secretion, multiple EVs could be formed from membrane fragments with a relatively larger size than the EVs produced by mechanism 1, most likely due to the absence of external force from the environment. Although this process in gram-positive bacteria appears similar to the explosive cell lysis in gram-negative bacteria, the outer-inner membrane vesicles, a form of EVs produced by gram-negative bacteria, were not observed in the gram-positive bacteria in this study. This is most likely due to the single layer of the cytoplasmic membrane in gram-positive bacteria. Furthermore, EV produced through mechanism 3 contained enterotoxin B as a virulence factor.

## Conclusions

Our findings highlight the diverse mechanisms for EV biogenesis in gram-positive bacteria, which have been overlooked in previous studies. Based on our nanoscale observations using various super-resolution microscopy techniques, we propose three major mechanisms of EV biogenesis through membrane blebbing (mechanisms 1 and 2) and explosive cell lysis (mechanism 3), which are similar to the mechanisms observed in gram-negative bacteria, albeit with some notable differences. These techniques shed light on the molecular mechanisms of EV biogenesis by imaging other EV constituents, such as genes, virulence factors, and cellular metabolites. Furthermore, the live-cell super-resolution imaging of EV biogenesis in gram-positive bacteria as a future work is useful for visualizing EV formation in gram-positive bacteria in real time, which will be needed to confirm the models proposed in this study. Overall, a better understanding of the molecular mechanisms of EV production in gram-positive bacteria will facilitate the development of therapeutics and biomedicine, including vaccines.

## Methods

### Sample preparation for fluorescence imaging

*S. aureus* (ATCC 12600) and *S. epidermidis* (ATCC 14790) were streaked from glycerol stocks onto tryptic soy agar (TSA) plates and grown overnight at 37°C. Then, a single colony was picked from a TSA plate and inoculated in tryptic soy broth (TSB) medium in a shaking incubator at 37°C and 200 rpm for 16 h. After inoculation, the fermented broth was centrifuged at 12,000 rpm for 2 min, and the supernatant was removed. The resulting pellet was resuspended in Dulbecco’s phosphate-buffered saline (DPBS) and fixed with 4% (v/v) paraformaldehyde (PFA; 15714; Electron Microscopy Sciences) in DPBS for 15 min at room temperature (RT). The fixed cells were then washed with DPBS and resuspended in freshly prepared 0.2% NaBH_4_ for 2 min at RT to reduce unreacted aldehyde groups. Finally, the samples were washed again with DPBS before centrifuging and labeling with fluorophores. The purified EVs were obtained from R&D Center of LG H&H. To purify EVs from *S. aureus (or S. epidermidis)*, EVs were isolated from cultured *S. aureus* (or *S. epidermidis*). They were then purified by ultracentrifugation at 10,000*g* for 30 min and 150,000*g* for 2.5 h (Hitachi, Chiyoda-ku, Tokyo, Japan). After ultracentrifugation, EV-rich pellets were diluted in distilled water to a final volume of 100 mL. The resuspended EV solution was further filtered with a 0.22 μm bottle-top filter, and the filtered EV solution was stored at 4°C. Purified EVs were confirmed by nanoparticle tracking analysis (NTA; Zetaview, Particle metrix, Germany), which is commonly used for EV identification. For example, the average diameter of purified EVs from *S. aureus* with a concentration of 3.0×10^9^ particles/mL was measured as 164.4 nm using NTA, which is consistent with the reported range for EVs from *S. aureus* (145 ± 15 nm) [34]. Also, the averaged diameter of the purified EVs from *S. epidermidis* with a concentration of 2.8×10^9^ particles/mL was measured as 206.8 nm from NTA, which is consistent with the reported range for the EVs from *S. epidermidis* (172 ± 17 nm )[34].

For Nile red dye labeling, pelleted bacteria were first diluted in DPBS and attached to a poly-L-lysine (PLL)-coated glass-bottom dish for 1 h at RT. After washing twice with DPBS, the cells were incubated in 10 nM Nile red membrane dye (415711000; Acros Organics) in DPBS for 30 min, followed by STORM imaging [35]. The Nile red-stained *S. aureus* prepared in bovine serum albumin (BSA)-treated coverslip also showed EVs similar to those in the sample prepared without BSA treatment, implying that the observed EVs does not result from the non-specific binding (Additional file 1: Fig. S14).

Alexa Fluor 647-conjugated WGA (WGA-AF647, W32466; Invitrogen) was used to visualize the cell wall. The centrifuged cells were incubated in WGA-AF647 for 60 min at RT, followed by a brief wash with DPBS. After removing the supernatant, the cells were diluted in DPBS and incubated in a PLL-coated glass-bottom dish for 1 h. Then, the dish was briefly washed twice in DPBS and imaged in imaging buffer for STORM, which was prepared with 100 mM cysteamine (30070; Sigma-Aldrich), 5% glucose (w/v), and oxygen-scavenging enzymes (0.5 mg/mL glucose oxidase [G2133; Sigma-Aldrich] and 38 μg/mL catalase [C3515; Sigma-Aldrich] in DPBS at pH 8.5).

For immunolabeling, the centrifuged cells were permeabilized in 0.2% Triton X-100 at RT for 20 min. After permeabilization, the pellet was incubated in a blocking buffer (3% [w/v] bovine serum albumin (BSA) in DPBS) for 30 min. The blocked cells were stained with the primary antibody in blocking buffer for 60 min at RT. Two types of *S. aureus* antibodies were used to detect enterotoxin B and protein A: PA1-7246 (Invitrogen) and ab20920 (Abcam), respectively. After washing twice with DPBS, the samples were labeled with Alexa Fluor 647-conjugated secondary antibody (A31573; Invitrogen) for 60 min at RT and then post-fixed with 2% PFA and 0.05% GA in DPBS for 10 min at RT. Finally, the bacterial cells were attached to a PLL-coated glass-bottom dish and incubated for 1 h, followed by a brief wash with DPBS. The samples were either stored in DPBS at 4°C or suspended in the STORM imaging buffer if they were to be immediately imaged.

For multi-color imaging of the peptidoglycan layer and cytoplasmic membrane using WGA-AF647/anti-protein A/anti-enterotoxin B and Nile red, the samples were first prepared as described for the procedure of WGA-AF647 labeling or immunolabeling of protein A/enterotoxin B. Next, the cells were attached to the PLL-coated gridded coverslip (P35G-1.5-14-CGRD-D; Mattek) for 1 h for STORM imaging. After the STORM imaging of WGA/protein A/enterotoxin B, the samples were washed twice with DPBS, incubated in 10 nM Nile Red solution in DPBS for 30 min, and imaged using STORM.

### STORM imaging

STORM imaging was performed using a custom-built inverted microscope (Ti2-U; Nikon) by adapting total internal reflection fluorescence illumination to reduce the background signal [36]. The sample was illuminated using an excitation laser with wavelengths of 561 nm (100 mW, OBIS; Coherent) and 647 nm (120 mW, OBIS; Coherent) through a ×100 oil immersion objective lens (1.49 NA; Olympus). Irradiation using a 405-nm (0.2–0.5 mW, OBIS; Coherent) laser was used for reactivation if needed. The fluorescence emission from the sample was filtered using a bandpass emission filter (LF 408/488/561/635-B; Semrock) and imaged using an EMCCD camera (iXon Ultra 888; Andor). A total of 50,000 frames were usually acquired at a frame rate of 60–100 Hz. 3D images were obtained by introducing a cylindrical lens into the image-detection pathway for astigmatism.

For multi-color imaging of Nile red and WGA-AF647/enterotoxin B/protein A, the red channel of the sample was imaged first by illumination with a 647-nm laser, and then the green channel of the sample for the same area was imaged by excitation with a 561-nm laser. Before imaging the sample, beads in the same region of interest were imaged in 647 and 561 channels to map different color channels. Then, WGA in the samples was first imaged with an excitation laser of 647 nm using a bandpass emission filter (LF 408/488/561/635-B; Semrock) to image the cell wall. Nile red images were then obtained with an excitation laser of 561 nm using a long pass filter (BLP02-561R-25) and a dichroic mirror (Di03-R561-t1-25x36). The gridded cover glass was used to obtain the same sample area.

The STORM images were reconstructed by fitting the point spread functions in each frame with a Gaussian function to determine their centroid positions. These collected centroids were drift-corrected and rendered using several parameters for the final STORM image. For multi-color images, the images from two color channels of the same sample area were correlated using custom-written MATLAB code [13].

### EM imaging

For SEM imaging, bacterial samples cultured in TSB were centrifuged at 12,000 rpm for 2 min, followed by resuspension in DPBS. The samples were then pelleted and fixed in 2.5% glutaraldehyde (GA) in DPBS overnight at 4°C. After washing, the fixed bacterial samples were spotted on silicon wafers. When the wafer was completely dried, the sample was dehydrated using a graded ethanol series (30%, 50%, 70%, 80%, 90%, and 100% v/v) diluted in distilled water for 20 min twice in each step. The dehydrated samples were dried overnight and coated with platinum prior to imaging. SEM images were obtained using an S-4800 field emission scanning electron microscope (Zeiss) at 15 keV.

For correlative STORM and SEM imaging, samples were prepared for each fluorescence imaging step and then imaged using STORM. After STORM imaging, the samples were washed thrice with distilled water. The washed samples were prepared for SEM imaging, as described above. The cover glass attached to the dish was separated from the dish by removing the adhesive with ethanol. The cover glass was then coated with platinum before the SEM imaging. The same area in the sample shown in the STORM images was identified in the SEM setup by finding the position marked with a diamond pen on the glass-bottom dish. The correlative STORM and SEM images were overlaid based on the features visualized in each image.

For TEM imaging, the bacterial sample grown in the TSB medium was centrifuged at 12,000 rpm for 2 min and then briefly washed with DPBS. After centrifugation, pelleted samples were fixed with 2.5% GA overnight at 4°C. After washing thrice with cacodylate buffer, the samples were post-fixed with 1% osmium tetroxide in 0.1 M cacodylate buffer for 60 min on ice. The samples were then washed twice with cacodylate buffer and dehydrated using a graded ethanol series. Fully dehydrated samples were infiltrated with Epon 812 resin using propylene oxide and embedded in 100% Epon 812 resin. Using an EM UC7 ultramicrotome (Leica, Austria), 60-nm thin ultrathin sections were obtained and collected on 100 mesh copper grids. Finally, the ultrathin sections were stained with uranyl acetate and lead citrate and imaged at 120 kV using the KBSI Bio-HVEM System (JEM-1400 Plus; JEOL, Tokyo, Japan).

### Image quantification

Quantification of EVs in EM images was performed using the ImageJ software. The diameter of individual EVs was measured from SEM and TEM images by using the “Straight” tool in ImageJ. The size of each EV was considered to be the average of the longest and shortest measured diameters. First, for comparison of the cell wall density for each mechanism shown in Fig. 3D, each EV observed from the multi-color STORM images was classified into different mechanisms based on the criteria described in the next section. The cell wall density was measured based on the relative distance from the center of each blebbing EV. The histogram generated from multiple EVs was fitted using one or multi-Gaussian functions for each mechanism to compare the cell wall density near the EV released site. For bacteria without EVs, the cell wall density was measured by counting the number of localized molecules along the cell wall from random positions. The histogram generated from multiple positions was then fitted with a multipeak curve.

Next, for the graph of the contrast and curvature of the cell wall shown in Fig. 3E, each EV observed from the TEM images was classified into different mechanisms based on the criteria described in the next section. The contrast and curvature of the cell wall were then measured at the EV budding site from the TEM images. The curvature measurement was performed using the Kappa curvature analysis plugin in ImageJ. By defining the points of the EV budding site along the cell wall, the corresponding B-spline curve was constructed using a point distance minimization algorithm. The curvature of this fitted line is defined as the curvature of the cell wall. Next, the contrast was obtained by subtracting the averaged grayscale value of the background near the cell wall from the average grayscale value of the cell wall. The curvature and contrast of each EV were measured at the same position. The scatter plot of the contrast and curvature of the cell wall for each EV biogenesis event shows the reverse relationship of the contrast and curvature of the EVs produced by mechanism 1 (relatively lower contrast and a higher curvature) and mechanism 3 (relatively higher contrast and lower curvature). A single-molecule localization histogram was used to quantify EVs in the STORM images. The transverse profile of cell wall density near the EV budding site was generated using a single-molecule localization histogram. The diameter of the EVs shown in the STORM images was measured from the full width at half maximum (FWHM) of the single-molecule localization histogram for each EV.

To categorize the EV biogenesis mechanisms from STORM images, multi-color STORM images were used to categorize the cells into each mechanism. Cells that showed local cell wall lysis at the EV budding site were categorized as mechanism 1. If the released EV was surrounded by the remaining peptidoglycan layer (labeled with WGA, anti-protein A, or anti-enterotoxin B), it was classified as mechanism 2. When the released EVs near bacteria were observed within the fragments of the cell membrane and cell wall derived from explosive cell lysis, they were classified as mechanism 3. The remaining cell membrane at the explosion site is relatively weaker, with low localization density for cells categorized as mechanism 3 compared to the cells categorized as mechanisms 1 and 2.

To categorize the EV biogenesis mechanisms from SEM images, the samples were categorized into each mechanism depending on correlative images with STORM to determine whether the cell wall or the cell membrane was labeled. If the EVs shown on the exterior of the SEM images were labeled only with the cell membrane in the STORM images, we categorized them as mechanism 1. In contrast, if they were labeled with both the cell membrane and cell wall in the STORM images, we classified them as mechanism 2. When EVs were observed together with fragments of the cell membrane and cell wall, and the low membrane density at the explosion site in both the SEM and STORM images, the EV was classified as mechanism 3.

For TEM images, the samples were categorized just by TEM images; the cell membrane and cell wall were clearly distinguishable in the TEM images. Bacteria that had EV blebbing through the cell membrane with high curvature and did not have the peptidoglycan layer were categorized into mechanism 1. When the budding EV is encapsulated by the cell wall, it is classified as mechanism 2. If EVs were found with debris of the cell membrane and cell wall resulting from explosive cell lysis, they were categorized into mechanism 3.

## Supplementary Information

**Additional file 1. **Combined PDF of Supplemental figures S1 – S14. **Figure S1.** The resolution evaluation of STORM imaging. **Figure S2.** Representative multi-color STORM images of *S. aureus*. **Figure S3.** Observation of EVs stained with various membrane dyes. **Figure S4.** Purified EVs from *S. aureus* and *S. epidermidis.***Figure S5.** Super-resolution images of EVs from *S. epidermidis.***Figure S6.** The diameter distribution of EVs. **Figure S7.** STORM and SEM images of EVs shown in Fig. 1E. **Figure S8.** Representative super-resolution images and production rate of extracellular vesicles (EVs) and EV precursors from gram-positive bacteria under different conditions. **Figure S9.** STORM images in green and red channels of *S. aureus* in Fig. 1A **Figure S10.** STORM and SEM images of *S. aureus* in Fig. 2E. **Figure S11.** STORM images in green and red channels of *S. aureus* with produced EVs after the explosive cell lysis event for the overlay images shown in Fig. 2G. **Figure S12.** STORM images in green and red channels of *S. aureus* with EVs for each mechanism shown in Fig. 3B. **Figure S13.** Representative correlative STORM and SEM images of *S. aureus* with EVs produced by each mechanism. **Figure S14.** Super-resolution images of *S. aureus*-secreting EVs prepared on coverslips with and without BSA treatment.

**Additional file 2: Movie 1.** 3D STORM image of Nile red-labeled *S. aureus* without EVs.

**Additional file 3: Movie 2.** 3D STORM image of Nile red-labeled *S. aureus* with EVs.

## Data Availability

The data that supports the findings of this study are available in the supplementary material of this article.

## Abbreviations

FWHM: Full width at half maximum
EV: Extracellular vesicles
STORM: Stochastic optical reconstruction microscopy
OMV: Outer membrane vesicle
LM: Light microscopy
EM: Electron microscopy
SEM: Scanning electron microscopy
TEM: Transmission electron microscopy
WGA: Wheat germ agglutinin
IWZ: Inner wall zone
TSA: Tryptic soy agar
TSB: Tryptic soy broth
DPBS: Dulbecco’s phosphate-buffered saline
PFA: Paraformaldehyde
GA: Glutaraldehyde
PLL: Poly-L-lysine

## References

[1] Deatherage BL, Cookson BT (2012). Membrane vesicle release in bacteria, eukaryotes, and archaea: a conserved yet underappreciated aspect of microbial life. Infect Immun.

[2] Brown L, Wolf JM, Prados-Rosales R, Casadevall A (2015). Through the wall: extracellular vesicles in Gram-positive bacteria, mycobacteria and fungi. Nat Rev Microbiol.

[3] Bishop D, Work E (1965). An extracellular glycolipid produced by Escherichia coli grown under lysine-limiting conditions. Biochem J.

[4] Knox K, Vesk M, Work E (1966). Relation between excreted lipopolysaccharide complexes and surface structures of a lysine-limited culture of Escherichia coli. J Bacteriol Res.

[5] Toyofuku M, Nomura N, Eberl L (2019). Types and origins of bacterial membrane vesicles. Nat Rev Microbiol.

[6] Manning AJ, Kuehn MJ (2011). Contribution of bacterial outer membrane vesicles to innate bacterial defense. BMC Microbiol.

[7] Biller SJ, Schubotz F, Roggensack SE, Thompson AW, Summons RE, Chisholm SW (2014). Bacterial vesicles in marine ecosystems. Science..

[8] Gujrati V, Kim S, Kim S, Min JJ, Choy HE, Kim SC, Jon S (2014). Bioengineered bacterial outer membrane vesicles as cell-specific drug-delivery vehicles for cancer therapy. ACS Nano.

[9] Kaparakis-Liaskos M, Ferrero RL (2015). Immune modulation by bacterial outer membrane vesicles. Nat Rev Immunol.

[10] van der Pol L, Stork M, van der Ley P (2015). Outer membrane vesicles as platform vaccine technology. Biotechnol J.

[11] Toyofuku M, Cárcamo-Oyarce G, Yamamoto T, Eisenstein F, Hsiao C, Kurosawa M (2017). Prophage-triggered membrane vesicle formation through peptidoglycan damage in Bacillus subtilis. Nat Commun.

[12] Brown L, Kessler A, Cabezas-Sanchez P, Luque-Garcia JL, Casadevall A (2014). Extracellular vesicles produced by the G ram-positive bacterium B acillus subtilis are disrupted by the lipopeptide surfactin. Mol Microbiol.

[13] Chung J, Jeong D, Kim G, Go S, Song J, Moon E, Huh YH, Kim D (2021). Super-resolution imaging of platelet-activation process and its quantitative analysis. Sci Rep.

[14] Go S, Jeong D, Chung J, Kim G, Song J, Moon E, Huh YH, Kim D (2021). Super-resolution imaging reveals cytoskeleton-dependent organelle rearrangement within platelets at intermediate stages of maturation. Structure..

[15] Jeong D, Kim D (2022). Recent developments in correlative super-resolution fluorescence microscopy and electron microscopy. Mol Cell.

[16] Hell SW, Wichmann J (1994). Breaking the diffraction resolution limit by stimulated emission: stimulated-emission-depletion fluorescence microscopy. Opt Lett.

[17] Betzig E, Patterson GH, Sougrat R, Lindwasser OW, Olenych S, Bonifacino JS, Davidson MW, Lippincott-Schwartz J, Hess HF (2006). Imaging intracellular fluorescent proteins at nanometer resolution. Science..

[18] Rust MJ, Bates M, Zhuang X (2006). Sub-diffraction-limit imaging by stochastic optical reconstruction microscopy (STORM). Nat Methods.

[19] Jeong D, Kim D (2022). Super-resolution fluorescence microscopy-based single-molecule spectroscopy. Bull Korean Chem Soc.

[20] Burton E, Yakandawala N, LoVetri K, Madhyastha M (2007). A microplate spectrofluorometric assay for bacterial biofilms. J Ind Microbiol Biotechnol.

[21] Kehoe MA (1994). Cell-wall-associated proteins in Gram-positive bacteria. New Comprehensive Biochemistry.

[22] Merino N, Toledo-Arana A, Vergara-Irigaray M, Valle J, Solano C, Calvo E (2009). Protein A-mediated multicellular behavior in Staphylococcus aureus. J Bacteriol Res.

[23] Danielsen EM, Hansen GH, Karlsdóttir E (2013). Staphylococcus aureus enterotoxins A− and B: binding to the enterocyte brush border and uptake by perturbation of the apical endocytic membrane traffic. Histochem Cell Biol.

[24] Tweten RK, Iandolo JJ (1983). Transport and processing of staphylococcal enterotoxin B. J Bacteriol Res.

[25] Kim D, Deerinck TJ, Sigal YM, Babcock HP, Ellisman MH, Zhuang X (2015). Correlative stochastic optical reconstruction microscopy and electron microscopy. PLoS One.

[26] Hauser M, Wojcik M, Kim D, Mahmoudi M, Li W, Xu K (2017). Correlative super-resolution microscopy: new dimensions and new opportunities. Chem Rev.

[27] Burnham JC, Hashimoto T, Conti S (1970). Ultrastructure and cell division of a facultatively parasitic strain of Bdellovibrio bacteriovorus. J Bacteriol Res.

[28] Zhou X, Halladin DK, Rojas ER, Koslover EF, Lee TK, Huang KC, Theriot JA (2015). Mechanical crack propagation drives millisecond daughter cell separation in Staphylococcus aureus. Science..

[29] Nagakubo T, Nomura N, Toyofuku M (2020). Cracking open bacterial membrane vesicles. Front Microbiol.

[30] Turnbull L, Toyofuku M, Hynen AL, Kurosawa M, Pessi G, Petty NK (2016). Explosive cell lysis as a mechanism for the biogenesis of bacterial membrane vesicles and biofilms. Nat Commun.

[31] Takahashi S, Mizuma M, Kami S, Nishida H (2020). Species-dependent protoplast enlargement involves different types of vacuole generation in bacteria. Sci Rep.

[32] Nelson D, Loomis L, Fischetti VA (2001). Prevention and elimination of upper respiratory colonization of mice by group A streptococci by using a bacteriophage lytic enzyme. Proc Natl Acad Sci U S A.

[33] Matias VR, Beveridge TJ (2006). Native cell wall organization shown by cryo-electron microscopy confirms the existence of a periplasmic space in Staphylococcus aureus. J Bacteriol Res.

[34] Zaborowska M, Vazirisani F, Shah FA, Firdaus R, Omar O, Ekström K, Trobos M, Thomsen P (2021). Immunomodulatory effects exerted by extracellular vesicles from Staphylococcus epidermidis and Staphylococcus aureus isolated from bone-anchored prostheses. Biomaterials..

[35] Moon S, Yan R, Kenny SJ, Shyu Y, Xiang L, Li W, Xu K (2017). Spectrally resolved, functional super-resolution microscopy reveals nanoscale compositional heterogeneity in live-cell membranes. J Am Chem Soc.

[36] Chung J, Jeong U, Jeong D, Go S, Kim D (2022). Development of a new approach for low-laser-power super-resolution fluorescence imaging. Anal Chem.

