# Supplementary material for: Visualizing extracellular vesicle biogenesis in gram-positive bacteria using super-resolution microscopy
Source: BMC Biol. 2022 Dec 5;20:270. doi: 10.1186/s12915-022-01472-3 (PMC9720944; doi:10.1186/s12915-022-01472-3)
Supplement: Supplementary file 1 — Additional file 1. Combined PDF of Supplemental figures S1 – S14. Figure S1. The resolution evaluation of STORM imaging. Figure S2. Representative multi-color STORM images of S. aureus. Figure S3. Observation of EVs stained with various membrane dyes. Figure S4. Purified EVs from S. aureus and S. epidermidis.Figure S5. Super-resolution images of EVs from S. epidermidis.Figure S6. The diameter distribution of EVs. Figure S7. STORM and SEM images of EVs shown in Fig. 1E. Figure S8. Representative super-resolution images and production rate of extracellular vesicles (EVs) and EV precursors from gram-positive bacteria under different conditions. Figure S9. STORM images in green and red channels of S. aureus in Fig. 1A Figure S10. STORM and SEM images of S. aureus in Fig. 2E. Figure S11. STORM images in green and red channels of S. aureus with produced EVs after the explosive cell lysis event for the overlay images shown in Fig. 2G. Figure S12. STORM images in green and red channels of S. aureus with EVs for each mechanism shown in Fig. 3B. Figure S13. Representative correlative STORM and SEM images of S. aureus with EVs produced by each mechanism. Figure S14. Super-resolution images of S. aureus-secreting EVs prepared on coverslips with and without BSA treatment. [file 12915_2022_1472_MOESM1_ESM.pdf]

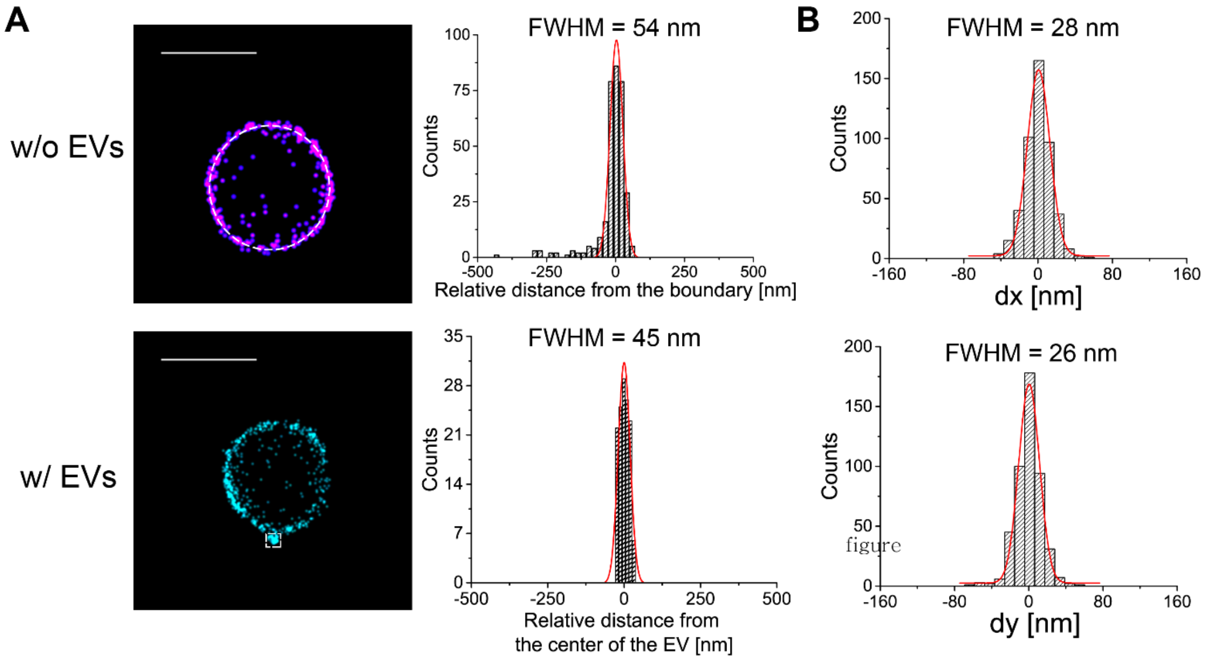

Fig. S1: The resolution evaluation of STORM imaging. **A** The xy cross-sections (left) of 3D STORM images of *S. aureus* without EVs (top) and with EVs (bottom) are shown in Fig. 1A. (Right top) The relative distance distribution with respect to the peptidoglycan layer fitted with an ellipse shows the nanoscale resolution of the imaging method when considering the thickness of the peptidoglycan layer. Gaussian fit gives a FWHM width of 54 nm. (Right bottom) Localization histogram of a released EV shown in Fig. 1A. Gaussian fit gives a FWHM width of 45 nm. Scale bars: 1 μm. **B** Statistics of positional variations in the locations of single Nile red molecules from Nile red-only sample without EVs. This shows the distribution histograms with FWHM values of 26–28 nm from the Gaussian fits for single Nile red molecules, implying the nanoscale super-resolution of Nile red imaging and no observation of Nile red precipitates under our staining condition.

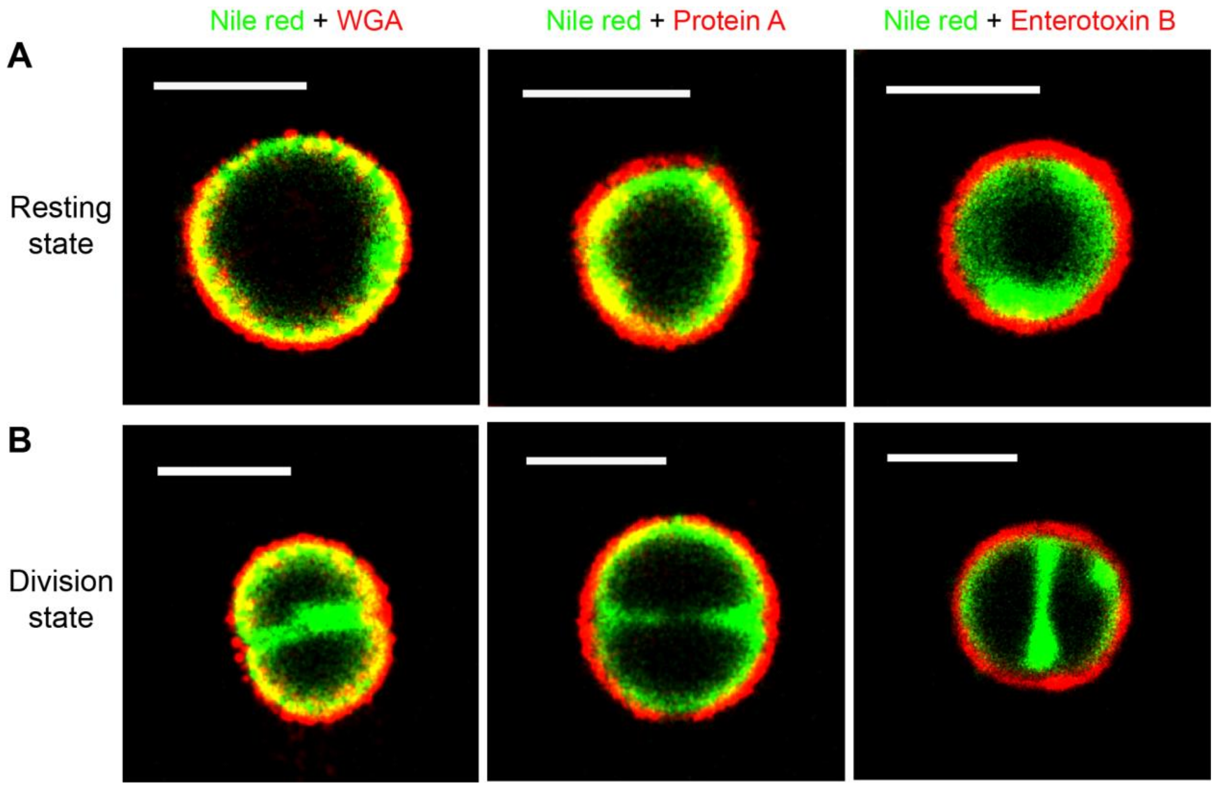

Fig. S2: Representative multi-color STORM images of *S. aureus* **A** in the resting state or **B** in the division state labeled with Nile red and WGA/anti-protein A/anti-enterotoxin B. The septum was labeled only by Nile red dye molecules. Scale bars: 1 μm.

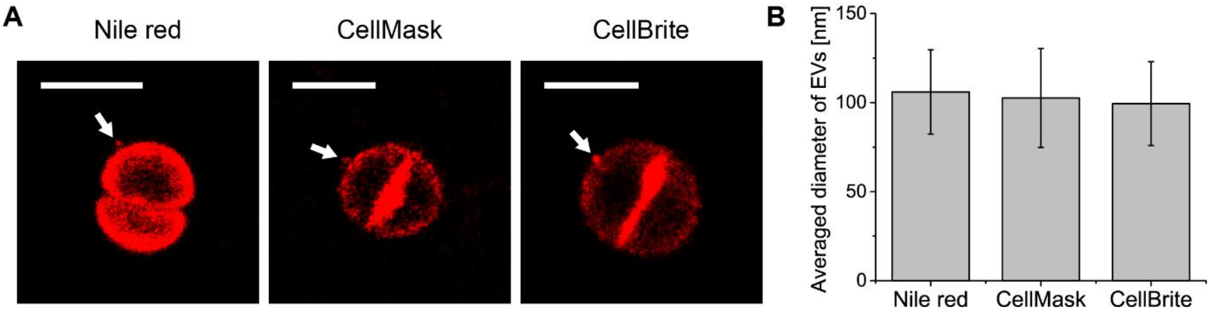

Fig. S3: Observation of EVs stained with various membrane dyes. **A** 3D STORM images of EVs labeled with Nile red (left), CellMask (middle), and CellBrite (right). **B** Size comparison of EVs observed from STORM images of EVs labeled with Nile red (left), CellMask (middle), and CellBrite (right). (mean±SD; n=31–75). Scale bars: 1 μm in **A**.

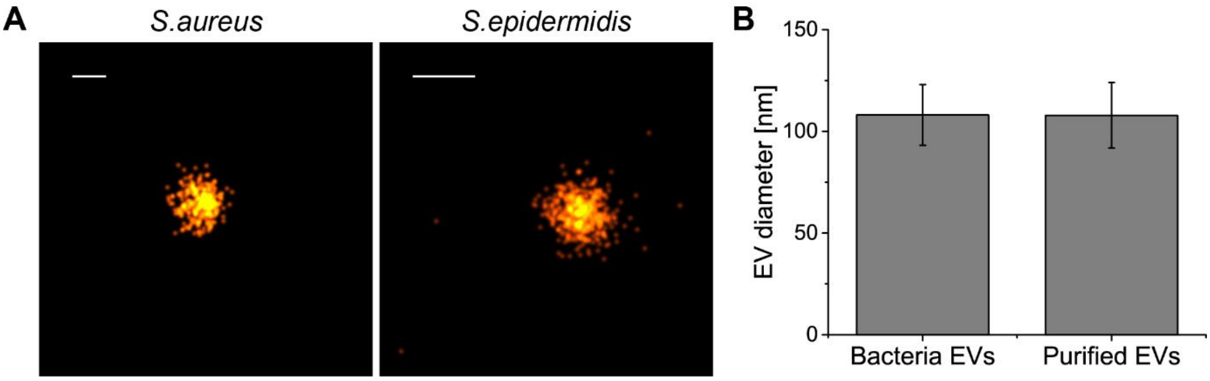

Fig. S4: Purified EVs from *S. aureus* and *S. epidermidis.* **A** 3D STORM images of purified EVs labeled with the Nile red from *S. aureus* (left) and *S. epidermidis* (right). **B** Size comparison of EVs observed from STORM images of Nile red-stained bacteria samples and purified EV samples. (mean±SD; n=100) Scale bars: 100 nm in **A**.

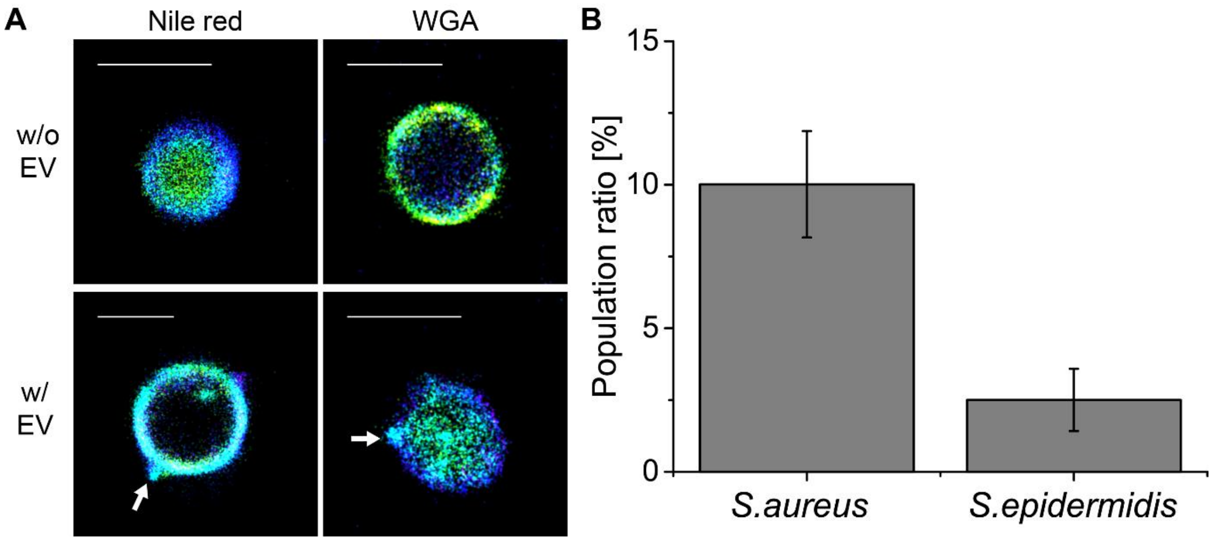

Fig. S5: Super-resolution images of extracellular vesicles (EVs) from *S. epidermidis*. **A** 3D STORM images of *S. epidermidis* without EVs (top) and with EVs (bottom). *S. epidermidis* were labeled with Nile red (left) or WGA (right). **B** Comparison of the population ratio of *S. aureus* and *S. epidermidis* secreting EVs. (mean±SD; n=600-700) Scale bars: 1 μm in **A**.

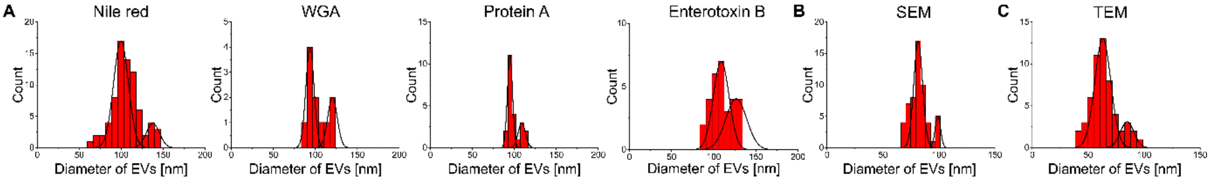

Fig. S6: The diameter distribution of EVs measured from **A** STORM, **B** SEM, and **C** TEM images. The histogram could be fitted with several Gaussian functions, implying the size heterogeneity of EVs. (n=11-71)

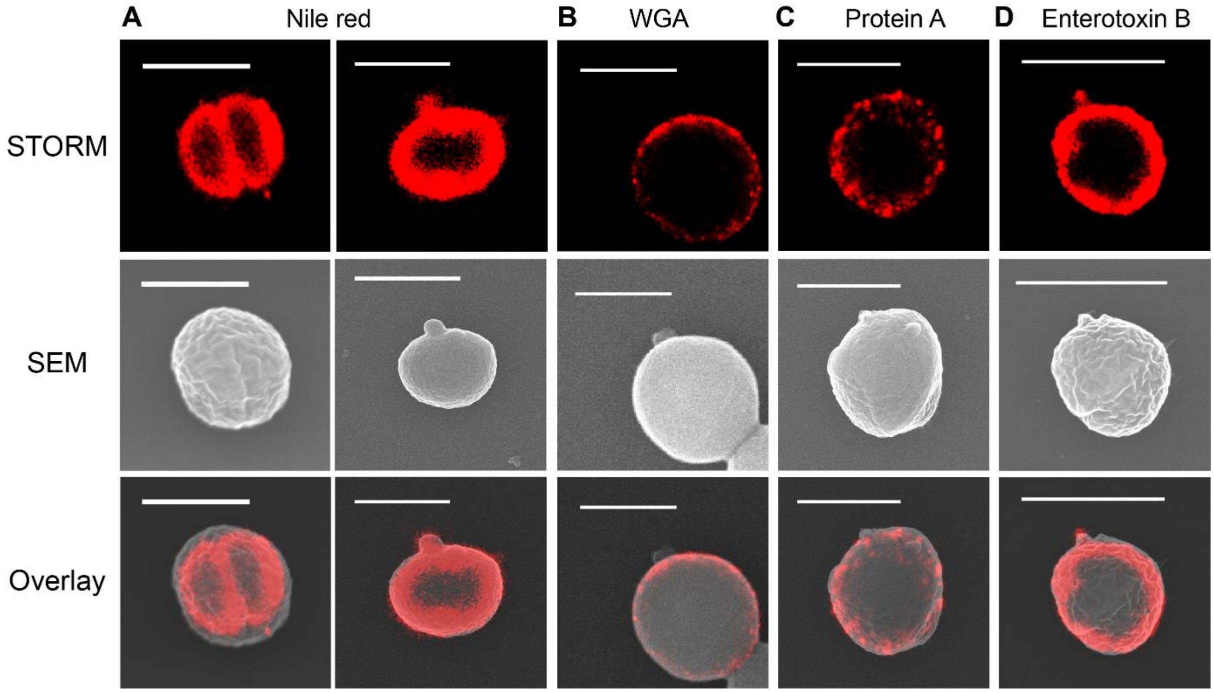

Fig. S7: STORM (top) and SEM (middle) images of EVs labeled with **A** Nile red, **B** WGA **C** anti-protein A or **D** anti-enterotoxin B for the overlay images (bottom) shown in Fig. 1E. Scale bars: 1 μm.

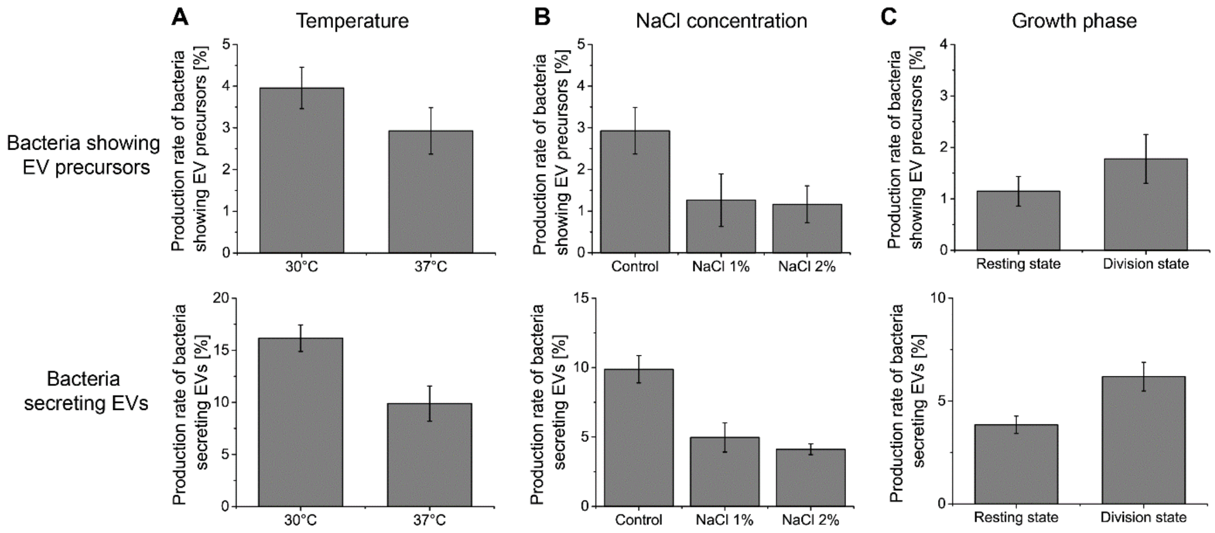

Fig. S8: Production rate of bacteria secreting extracellular vesicles (EVs) and bacteria showing membranous particles inside the peptidoglycan layer (i.e. EV precursors) from gram-positive bacteria under different conditions. The effects of **A** temperature, **B** NaCl concentration, and **C** growth phase on EV precursors (top) and the production of EVs (bottom) were investigated separately. Nile red staining was used to visualize EVs. The production rate was calculated as the population ratio of Nile red-labeled *S. aureus*-secreting EVs (or EV precursors). (mean±SD; n=430-660 for **A**, 450-1100 for **B**, 520-610 for **C**)

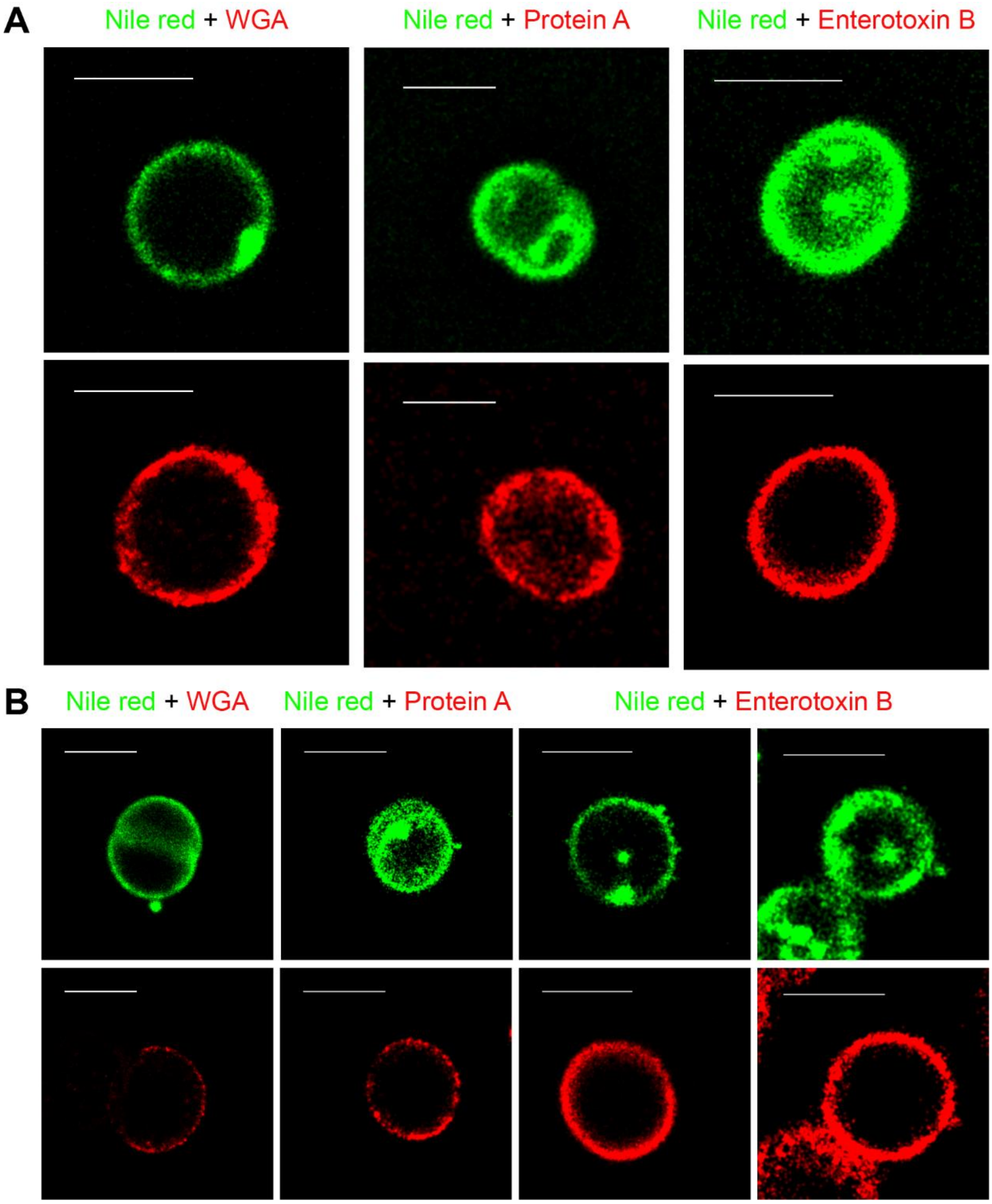

Fig. S9: STORM images in green (top) and red (bottom) channels of *S. aureus* **A** without EVs and **B** with EVs shown in Fig. 2A. Red: WGA, protein A, or enterotoxin B. Green: Nile red. Scale bars: 1 μm.

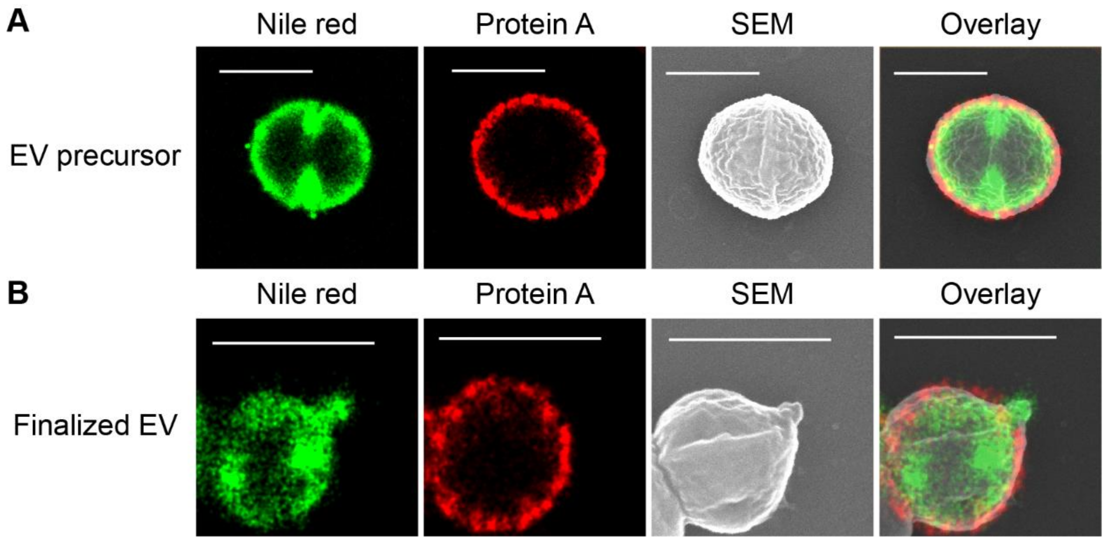

Fig. S10: STORM and SEM images of *S. aureus* with **A** EV precursor and **B** finalized EV for the overlay images shown in Fig. 2E. Scale bars: 1 μm.

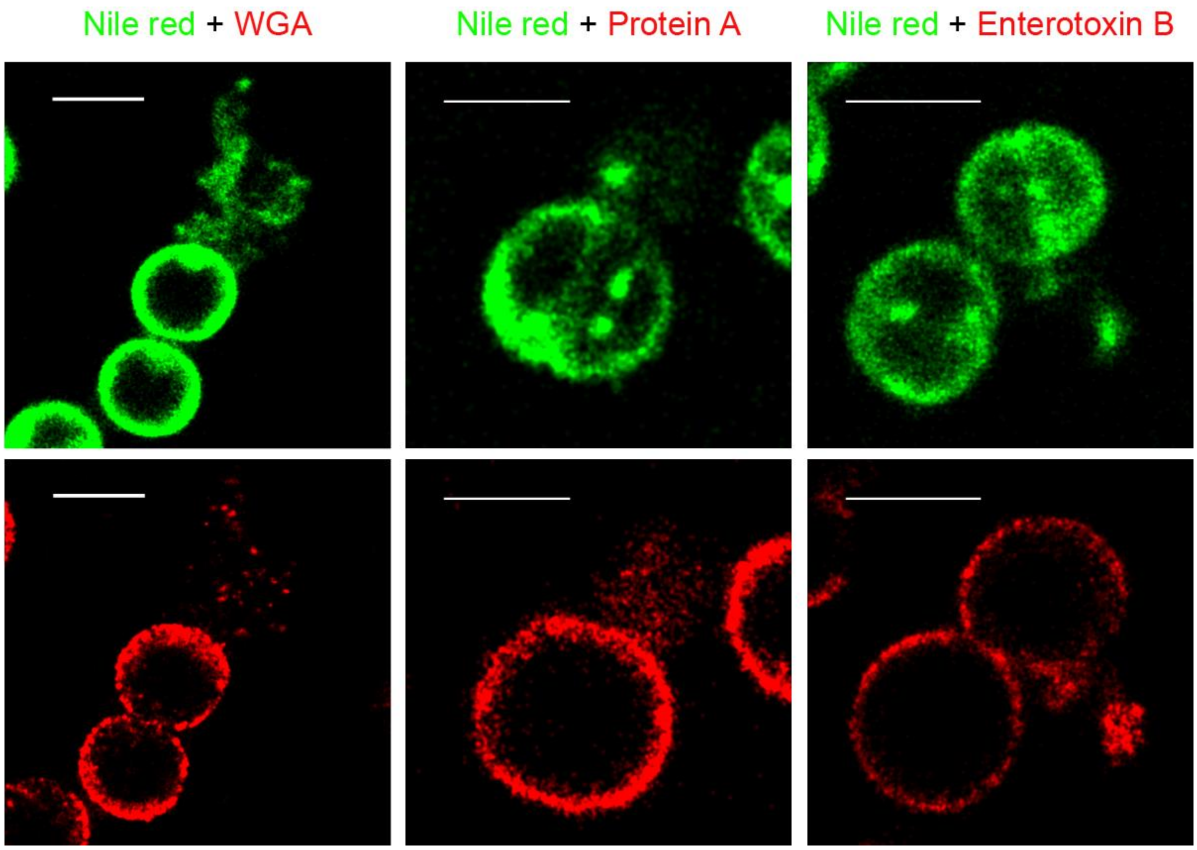

Fig. S11: STORM images in green (top) and red (bottom) channels of *S. aureus* with produced EVs after the explosive cell lysis event for the overlay images shown in Fig. 2G. Red: WGA, protein A, or enterotoxin B. Green: Nile red. Scale bars: 1 μm.

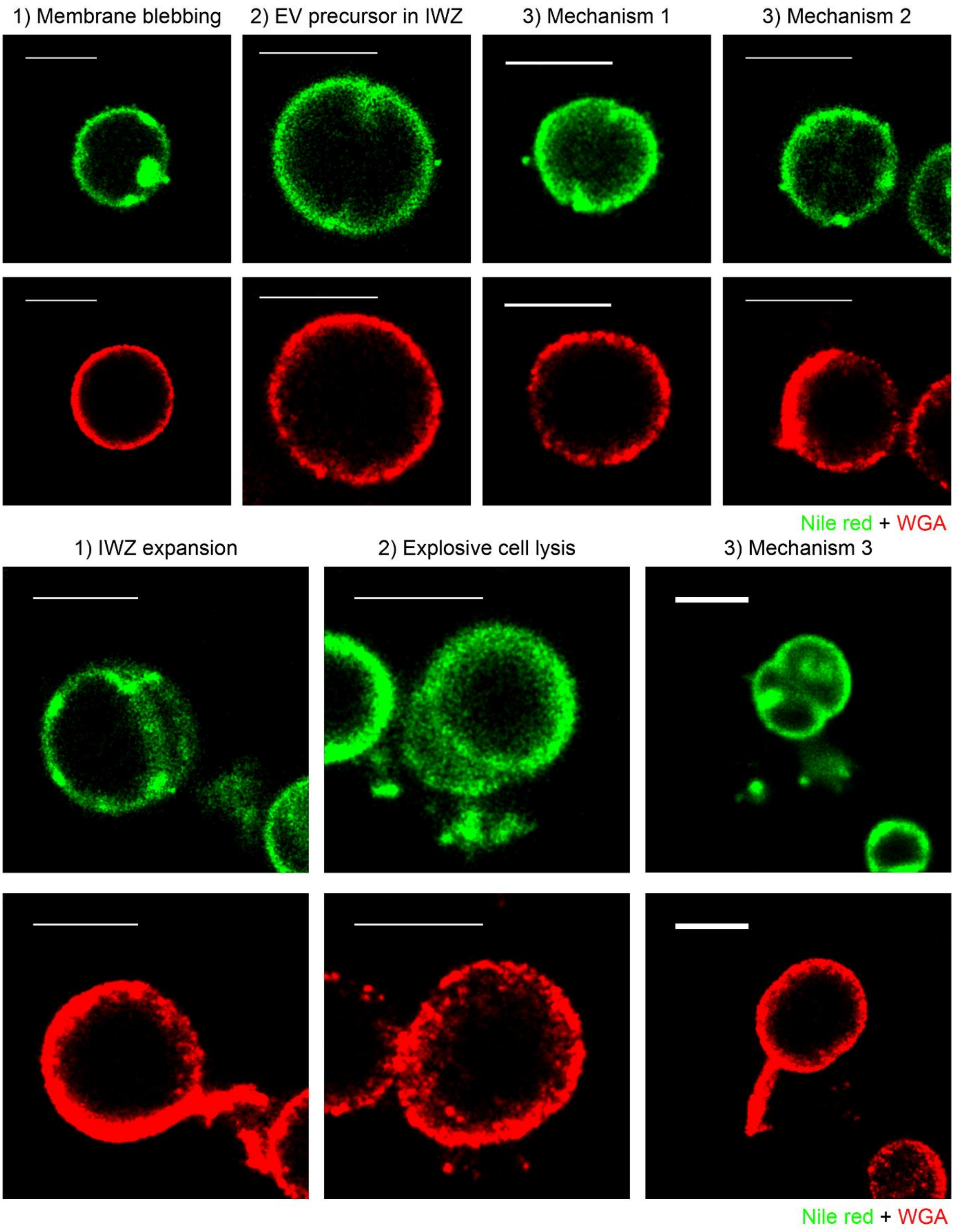

Fig. S12: STORM images in green (top) and red (bottom) channels of *S. aureus* with EVs for each mechanism shown in Fig. 3B. Red: WGA. Green: Nile red. Scale bars: 1 μm.

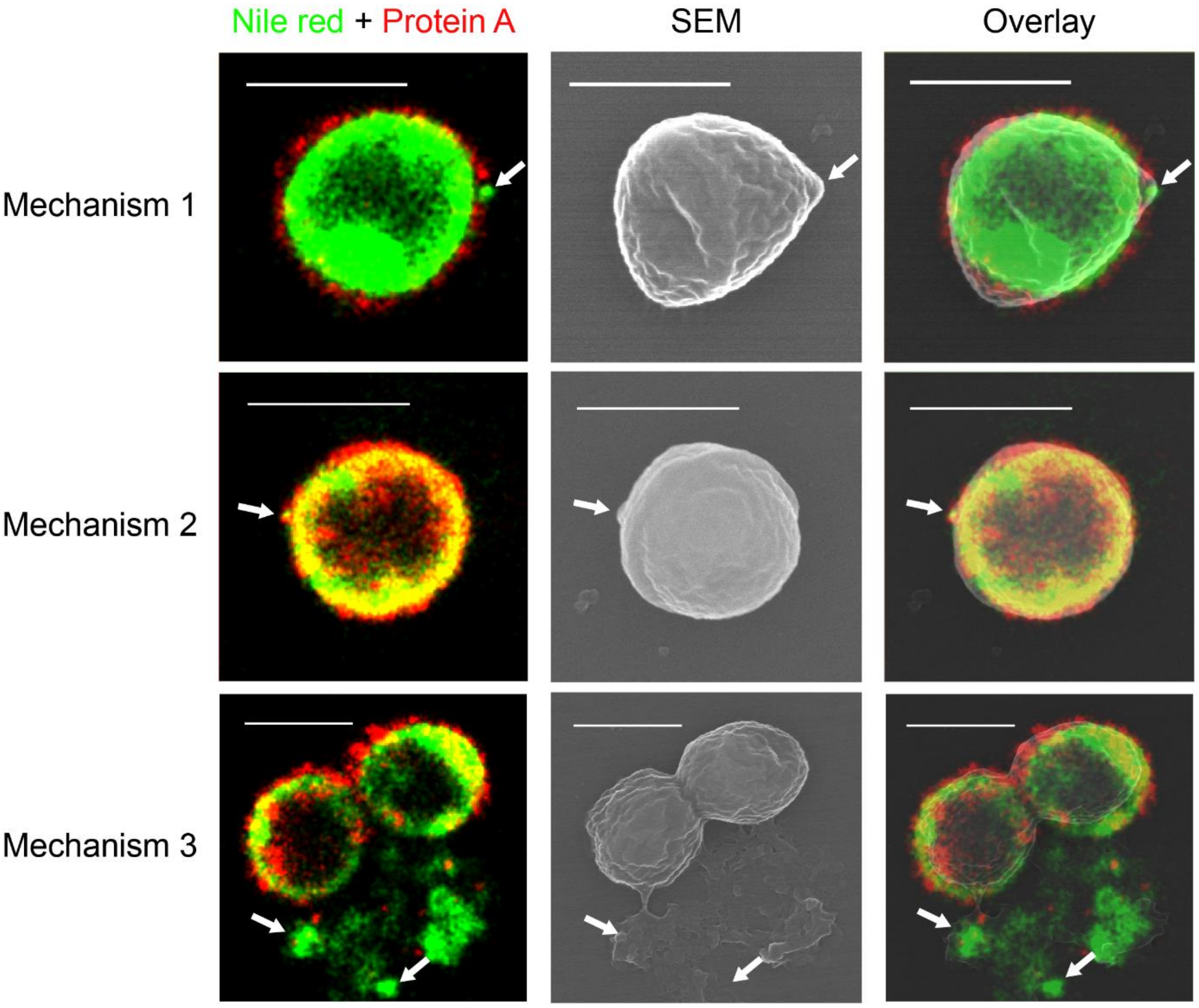

Fig. S13: Representative correlative STORM and SEM images of *S. aureus* with EVs produced by each mechanism. Left : multi-color STORM images. Middle: SEM images. Right: overlay images. Red: Protein A. Green: Nile red. White arrow: EV. Scale bars: 1 μm

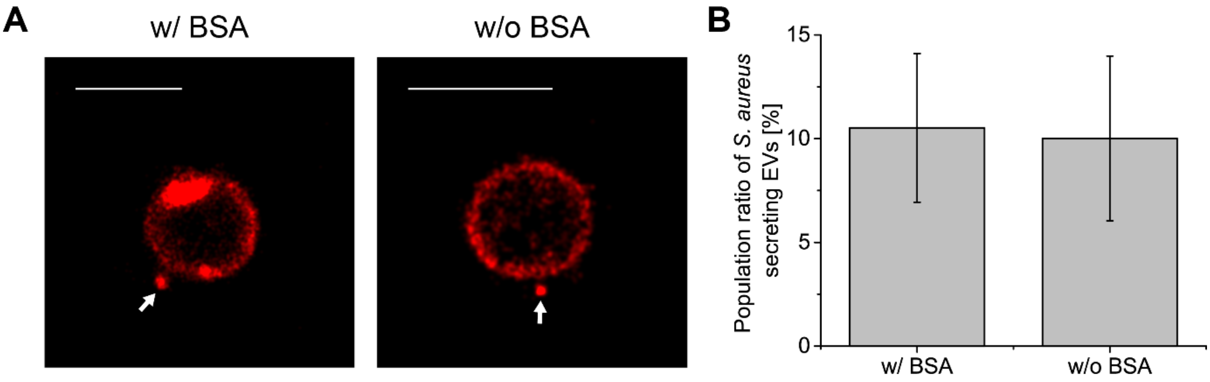

Fig. S14: Super-resolution images of *S. aureus*-secreting EVs prepared on coverslips with and without BSA treatment. **A** 3D STORM images of *S. aureus* prepared on coverslips with (left) and without (right) BSA treatment. **B** Comparison of the population ratio of *S. aureus*-secreting EVs prepared on coverslips, with and without BSA treatment. (mean±SD; n=339-709) Scale bars: 1 μm in **A**.
